# An efficient and improved method for virus-induced gene silencing in sorghum

**DOI:** 10.1186/s12870-018-1344-z

**Published:** 2018-06-18

**Authors:** Dharmendra Kumar Singh, Hee-Kyung Lee, Ismail Dweikat, Kirankumar S. Mysore

**Affiliations:** 1 Noble Research Institute, Ardmore, Oklahoma 73401 USA; 2 Department of Agronomy and Horticulture, University of Nebraska-Lincoln, Lincoln, Nebraska 68583 USA

## Abstract

**Background:**

Although the draft genome of sorghum is available, the understanding of gene function is limited due to the lack of extensive mutant resources. Virus-induced gene silencing (VIGS) is an alternative to mutant resources to study gene function. This study reports an improved and efficient method for *Brome mosaic virus* (BMV)-based VIGS in sorghum.

**Methods:**

Sorghum plants were rub-inoculated with sap prepared by grinding 2 g of infected *Nicotiana benthamiana* leaf in 1 ml 10 mM potassium phosphate buffer (pH 6.8) and 100 mg of carborundum abrasive. The sap was rubbed on two to three top leaves of sorghum. Inoculated plants were covered with a dome to maintain high humidity and kept in the dark for two days at 18 °C. Inoculated plants were then transferred to 18 °C growth chamber with 12 h/12 h light/dark cycle.

**Results:**

This study shows that BMV infection rate can be significantly increased in sorghum by incubating plants at 18 °C. A substantial variation in BMV infection rate in sorghum genotypes/varieties was observed and BTx623 was the most susceptible. *Ubiquitin* (*Ubiq*) silencing is a better visual marker for VIGS in sorghum compared to other markers such as *Magnesium Chelatase subunit H* (*ChlH*) and *Phytoene desaturase* (*PDS*). The use of antisense strand of a gene in BMV was found to significantly increase the efficiency and extent of VIGS in sorghum. *In situ* hybridization experiments showed that the non-uniform silencing in sorghum is due to the uneven spread of the virus. This study further demonstrates that genes could also be silenced in the inflorescence of sorghum.

**Conclusion:**

In general, sorghum plants are difficult to infect with BMV and therefore recalcitrant to VIGS studies. However, by using BMV as a vector, a BMV susceptible sorghum variety, 18 °C for incubating plants, and antisense strand of the target gene fragment, efficient VIGS can still be achieved in sorghum.

**Electronic supplementary material:**

The online version of this article (10.1186/s12870-018-1344-z) contains supplementary material, which is available to authorized users.

## Background

A current challenge to plant scientists is to increase plant productivity in the changing environment with limited resources such as water and fertile land. Sorghum [*Sorghum bicolor* (L.) Moench.] can grow in dry climatic conditions where summer temperatures are above 20°C [1, 2]. Sorghum is a C4 annual crop in the grass family and is characterized by its high photosynthetic efficiency. Grain, sweet, and forage type sorghums are all compatible with current agricultural production systems. Sorghum is the fifth most important cereal crop after rice, wheat, maize, and barley [2], with an annual production of about 65.5 million tons from the planted area of 45 million ha worldwide [3]. Sorghum is grown for food, feed for livestock and fuel. It is a food source for 500 million people in 30 countries [2]. Nearly 80% of the sorghum-growing area is in developing countries [2]. However, according to Food and Agriculture Organization of the United Nations, in 2014 the USA was the largest producer of sorghum followed by Mexico, Nigeria, Sudan and India [3]. Sorghum production is affected by nearly 150 species of pests, fungus, viruses and parasitic weeds [2]. Also, sorghum is exposed to a variety of abiotic stresses because it is mainly grown on marginal lands [2]. The study of gene function in sorghum will lead to increased productivity of sorghum by improving plant productivity and resistance to biotic and abiotic stresses.

The whole genome sequence of sorghum (BTx623) is available to identify gene sequences that control desirable traits and facilitate molecular breeding (www.phytozome.net/sorghum) [4, 5]. However, it is critical to validate the predicted role of the genes. Only a few mutant collections are available for sorghum: a large-scale ethyl methanesulfonate mutagenized library that has nearly 5,000 lines [6] and 5,466 gamma-ray-induced mutant M2 lines [7]. Most of these lines are uncharacterized and therefore the understanding of gene function in sorghum is far from complete.

Virus-induced gene silencing (VIGS) is a quick and robust method to assess the function of a gene or multiple genes by transient post-transcriptional gene silencing [8]. The VIGS mechanism relies on plant antiviral defenses involved in the degradation of viral RNA [9, 10]. For VIGS, a fragment of a plant gene is inserted into the viral genome. Small interfering (si) RNAs specific to the viral genome and inserted plant gene sequences are generated by Dicer-like protein (DCL). The siRNA integrates with RNA-induced silencing complex (RISC) and targets the viral RNA and specific plant mRNA for degradation [11].

The two critical factors for the success of VIGS are; 1) the ability of the virus to infect the plant, 2) the ability of the plant to defend itself from the virus with limited effect on its growth and development. Several VIGS vector systems are available to silence genes in dicotyledons and monocotyledons [8, 12–14]. For monocotyledons, different VIGS vector systems have been developed based on *Brome mosaic virus* (BMV; [15]), *Barley stripe mosaic virus* (BSMV;[16, 17]), *Bamboo mosaic virus* [18], *Cymbidium mosaic virus* [19], *Cucumber mosaic virus* [20], *Foxtail mosaic virus* [21, 22], and *Rice tungro bacilliform virus* [23].

We tested two VIGS vector systems, BMV and BSMV, to silence genes in sorghum. Ding et al. [15] developed BMV based VIGS vector to silence genes in monocots. Several studies have used BMV based VIGS to silence genes in barley [15], maize [15], rice [15], *Festuca arundinacea* (Tall Fescue) [24], and sorghum [25, 26]. However, the reported BMV based method for silencing a gene using VIGS in sorghum is cumbersome and is not very efficient with very low frequency of silencing. BSMV was used to silence genes in barley [16, 27], wheat [27, 28] and in *Nicotiana benthamiana* [29] but not reported to silence genes in sorghum. Here, we report an improved method to efficiently silence a gene in sorghum using VIGS. We compared several parameters and identified the best environment condition, marker gene, VIGS vector, and sorghum variety for efficient VIGS in sorghum.

## Results

### An improved, simple and efficient method to deliver virus into sorghum

**Fig. 1:**
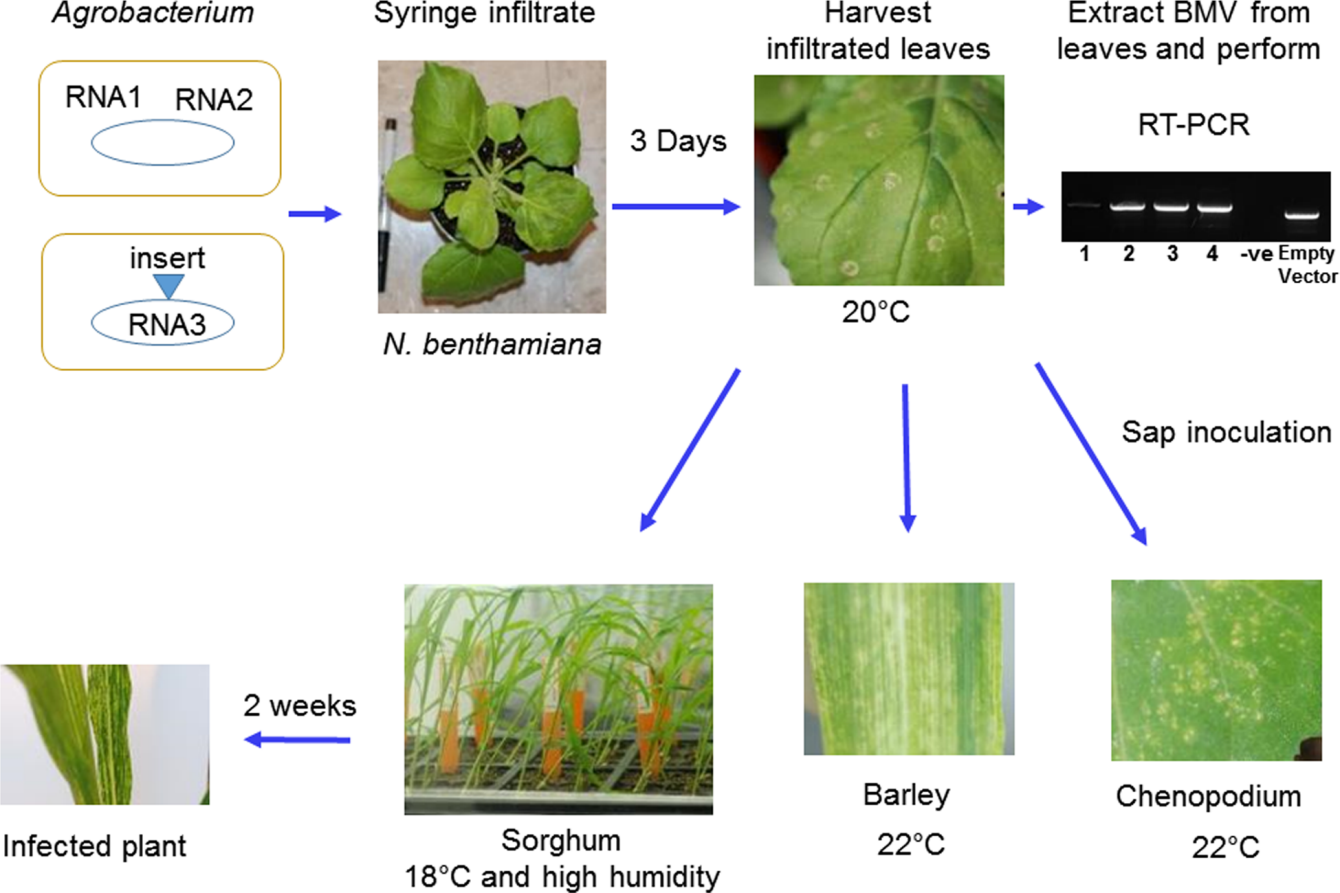
Schematic representation of BMV based VIGS in sorghum. For VIGS, the BMV genome is cloned into two *Agrobacterium* strains (see Zhu et al. 2014). One *Agrobacterium* strain has RNA1 and RNA2 of BMV, and another strain has modified RNA3 with restriction sites to insert plant gene fragments for silencing. The two *Agrobacterium* strains are multiplied to 1.5 OD_600_ and mixed in equal amounts to infiltrate *N. benthamiana* to reconstitute and multiply the virus. The virus is extracted from *N. benthamiana* leaves. RT-PCR is performed using viral RNA as template and primers flanking the insert to confirm the presence of gene fragment. The sap of infected *N. benthamiana* is used to inoculate the plants with BMV. The inoculated *Chenopodium* and barley plants can be kept at 22 °C, whereas inoculated sorghum plants should be maintained at 18 °C

BMV-based VIGS vector [15] has been successfully used to silence genes for functional characterization in monocots such as maize [15, 30, 31], barley [15], and rice [15]. The previous method [25, 26] used for VIGS in sorghum was not very efficient in our laboratory and needs *in vitro* RNA synthesis, which is cumbersome and expensive. Here, an easier and an inexpensive method using *Agrobacterium*-based BMV-VIGS vectors [32, 33] was used to silence genes in sorghum. The BMV genome is made of three positive strand RNAs (RNA 1 [3.2 kb], RNA 2 [2.9 kb] and RNA 3 [2.1 kb]). For VIGS, RNA 1 and RNA 2 are cloned into one *Agrobacterium* binary vector and RNA 3 into another [32]. A fragment of a plant gene was inserted into RNA 3 as described earlier [15]. We adopted a method that was used earlier to do BMV based VIGS in maize [33]. Briefly, the two *Agrobacterium* strains containing RNA 1, RNA 2, and RNA 3 were grown to 1.5 O.D_600_ and mixed in equal amounts to infiltrate into leaves of three weeks old *N. benthamiana* plant for viral multiplication (Fig. 1). The infected *N. benthamiana* leaves were harvested four days after infection and either the sap was extracted or stored at -80 °C. The sap of the infected *N. benthamiana* was used to inoculate *Chenopodium*, barley and sorghum plants.

### Tolerance of sorghum to BMV decreases at lower temperature

**Fig. 2:**
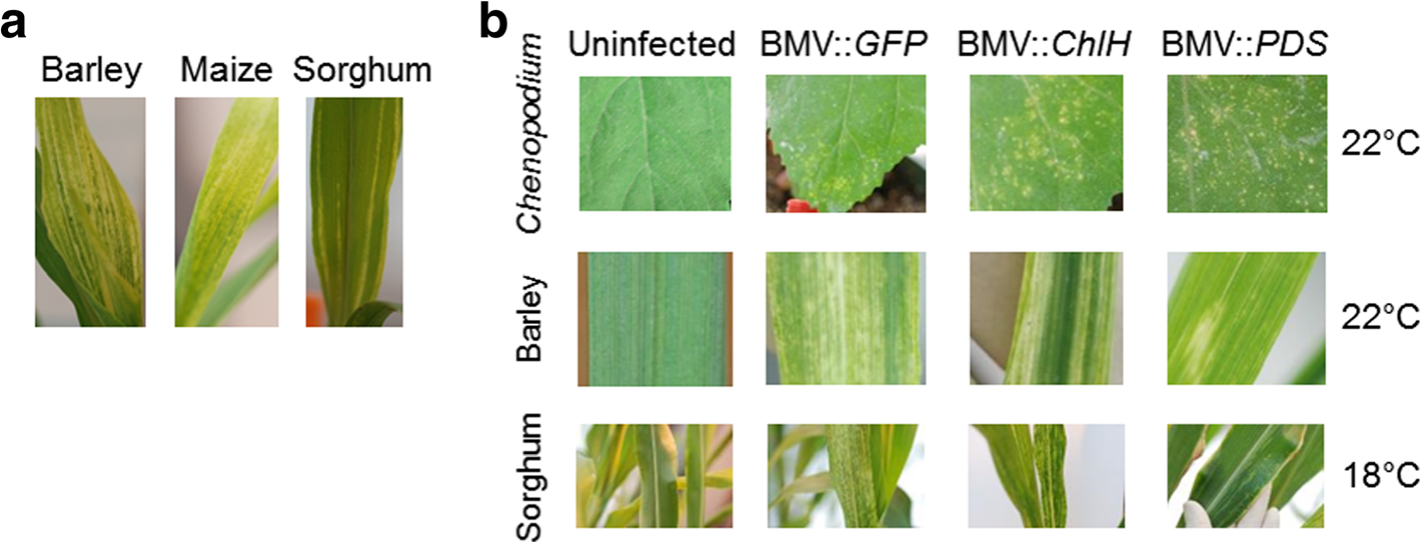
Sorghum plants incubated at 18 °C enhance BMV infection. **a** BMV::*GFP* infection showed more viral symptoms (bleaching/yellowing) in maize and barley compared to sorghum at 22 °C. **b** Virus with *GFP* and sorghum gene fragments: *ChlH*, or *PDS*, were grown in *N. benthamiana*, and sap was isolated and rubbed on *Chenopodium*, barley, and sorghum plants. BMV was able to infect *Chenopodium* and barley at 22 °C and sorghum at 18 °C.

An important factor for successful VIGS is the ability of the virus to infect and spread within the plant. Contrary to barley and maize, in our greenhouse conditions, we were not able to see visible viral symptoms at high frequency in sorghum after BMV infection at 22 °C (Fig. 2a). BMV-*GFP* inoculated maize and barley developed extensive white stripes on the systemic leaves at 22 °C (Fig. 2a). *GFP* sequence does not have homology to plant DNA and therefore will not cause any gene silencing in plants. White or yellow stripes are a common symptom due to BMV infection. However, only ~10% of the inoculated sorghum (BTx623 line) plants develop limited white stripes on systemic leaves at 22 °C. Further, inoculation with BMV VIGS construct with a fragment from *GFP* or *SbChlH*, or *SbPDS* genes were not able to produce any viral symptoms or gene silencing in sorghum at 22 °C. In this study, *GFP* gene fragment was used as a negative control and there are no plant sequences that are homologous to *GFP*. These constructs produced viral symptoms when infected on *Chenopodium* and barley at 22 °C suggesting that the constructs were infectious (Fig. 2b). Interestingly, all the BMV-inoculated BTx623 sorghum plants (100%) developed disease symptoms when kept at 18 °C for three days prior to BMV inoculation and three weeks post-BMV inoculation (Fig. 2b). The extent of infection symptoms varied among sorghum plants. These results suggest that sorghum plants incubated at low temperature (18 °C) show better symptoms after BMV challenge than those at 22 °C. Despite viral symptoms at 18 °C, the sorghum plants did not show any visual *ChlH* or *PDS* silencing symptoms.

### *Ubiquitin* is a better marker for VIGS in sorghum

**Fig. 3:**
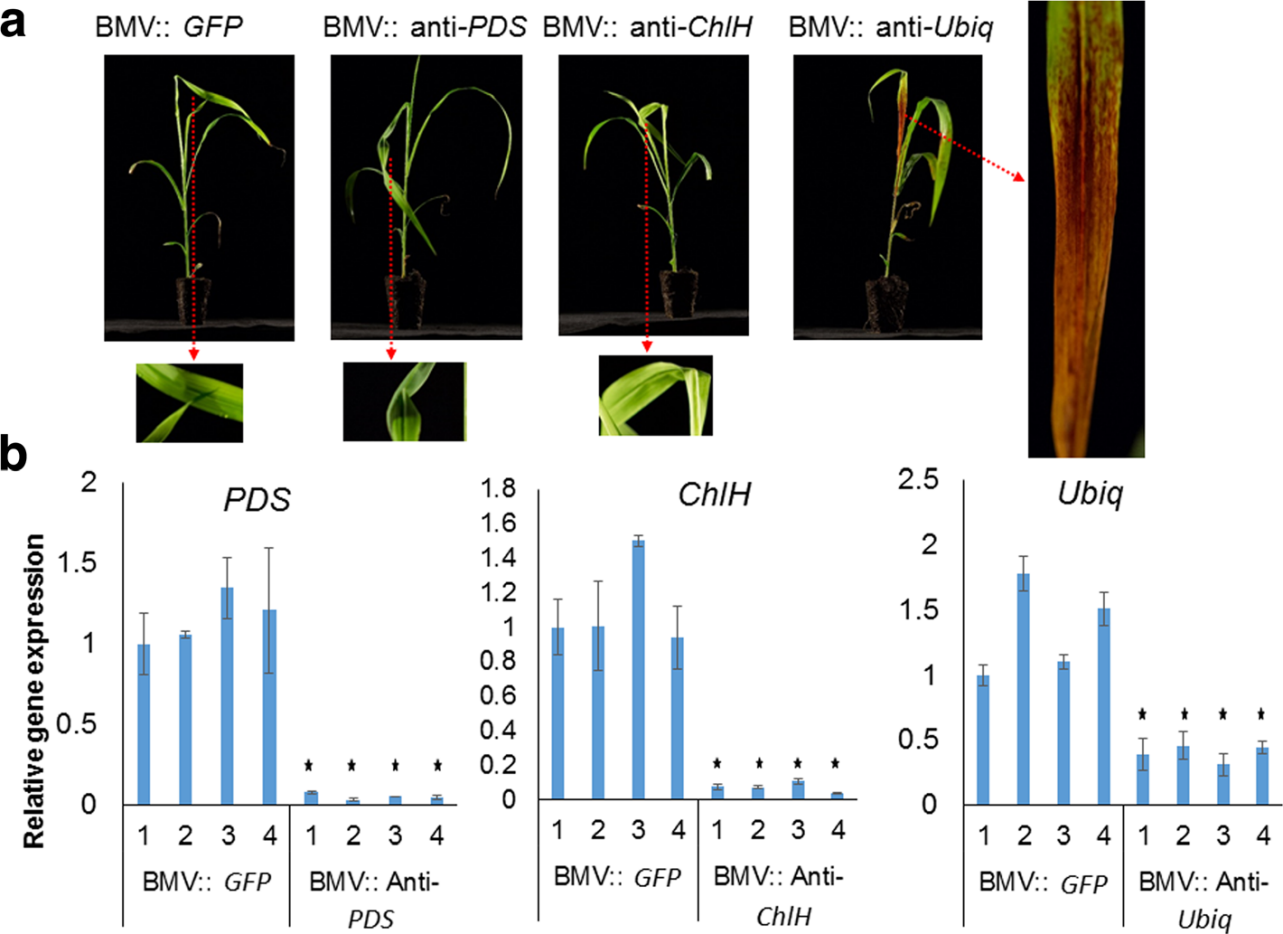
*Ubiquitin* silencing is a better marker for VIGS in sorghum. Sorghum plants were rub inoculated with BMV VIGS constructs to silence *ChlH*, *PDS,* or *Ubiq* genes. **a** Only BMV::anti-*Ubiq* inoculated plants had a visible phenotype that is different than the control (BMV::*GFP*). Each viral vector was rub inoculated on 10 sorghum seedlings. Similar results were observed in all the seedlings. A representative image is shown here. **b** The RNA was extracted from the second leaf above inoculated leaf of sorghum for gene expression analysis using qRT-PCR. The expression of *PDS*, *ChlH*, and *Ubiq* was significantly lower in the BMV::anti-*PDS*, BMV::anti-*ChlH*, and BMV::anti-*Ubiq*, respectively, compared to control plants (BMV::*GFP*). Error bars indicate standard deviation of three replicates. The sample was analyzed by student’s *t* test. Asterisks indicate a statistically significant difference compared with the control at P ≤ 0.01

The *Phytoene desaturase* (*PDS*) and *Magnesium Chelatase subunit H* (*ChlH*) silencing are popularly used as visible markers for VIGS-mediated gene silencing in many plant species [29, 34]. An anti-sense strand of the sorghum *ChlH* or *PDS* gene fragment was used in this study to test the efficiency of BMV-mediated VIGS in sorghum (Fig. 3). Upon inoculation of these constructs to sorghum, at 18 °C, we clearly observed viral symptoms (white stripes) suggesting successful infection in sorghum. Surprisingly, we did not notice whitening of leaves due to *PDS* silencing in the BMV::anti-*PDS* inoculated plants or yellowing of leaves due to *ChlH* silencing in the BMV::anti-*ChlH* inoculated plants (Fig. 3a). However, the real-time quantitative RT-PCR (qRT-PCR) data distinctly showed a reduction of transcript level for *ChlH* or *PDS* genes in BMV::anti-*ChlH* or BMV::anti-*PDS* inoculated plants, respectively (Fig. 3b). A weak silencing symptom may be masked because the white stripes observed due to the BMV::*GFP* viral infection are quite similar to *PDS* or *ChlH* silencing symptoms. Therefore, we concluded that *PDS* or *ChlH* gene silencing are not good visual markers to measure the efficiency of gene silencing in sorghum.

To identify a better visual marker for VIGS in sorghum plants, we developed a BMV-VIGS construct with a fragment of *Ubiquitin* (*Ubiq*) gene. We chose to silence *Ubiq* because it is a crucial protein for plant development and silencing this gene was expected to result in a visual pehnotype. Partial reduction of *Ubiq* transcripts by transient gene silencing in barley has been shown to cause cell death [35]. Except for the *GFP* (control) all the gene constructs used were able to silence the target genes when examined by qRT-PCR (Fig. 3b). However, the visible phenotype due to silencing was observed only in the *Ubiq* silenced plants that showed browning (Fig. 3a). Therefore, we concluded that among all the different silencing markers used, *Ubiq* is the best visual marker for VIGS in sorghum.

### Antisense strand of *Ubiq* is more efficient in VIGS than the sense strand

Inserting the antisense strand of a gene in the BMV VIGS vector resulted in a greater degree of silencing compared to using a sense strand in barley [36, 37]. In other studies, the sense or antisense strand of a gene resulted in a similar level of BSMV-based VIGS in barley and wheat [17, 38]. This prompted us to compare the VIGS efficiency of sense versus antisense strand. We generated VIGS construct using sense or antisense of the sorghum *Ubiq* gene. The same 206 bp fragment of sorghum *Ubiq* gene was selected to develop both sense and anti-sense constructs. The sequence selected had less homology to other *Ubiq* gene family members of sorghum.

**Fig. 4:**
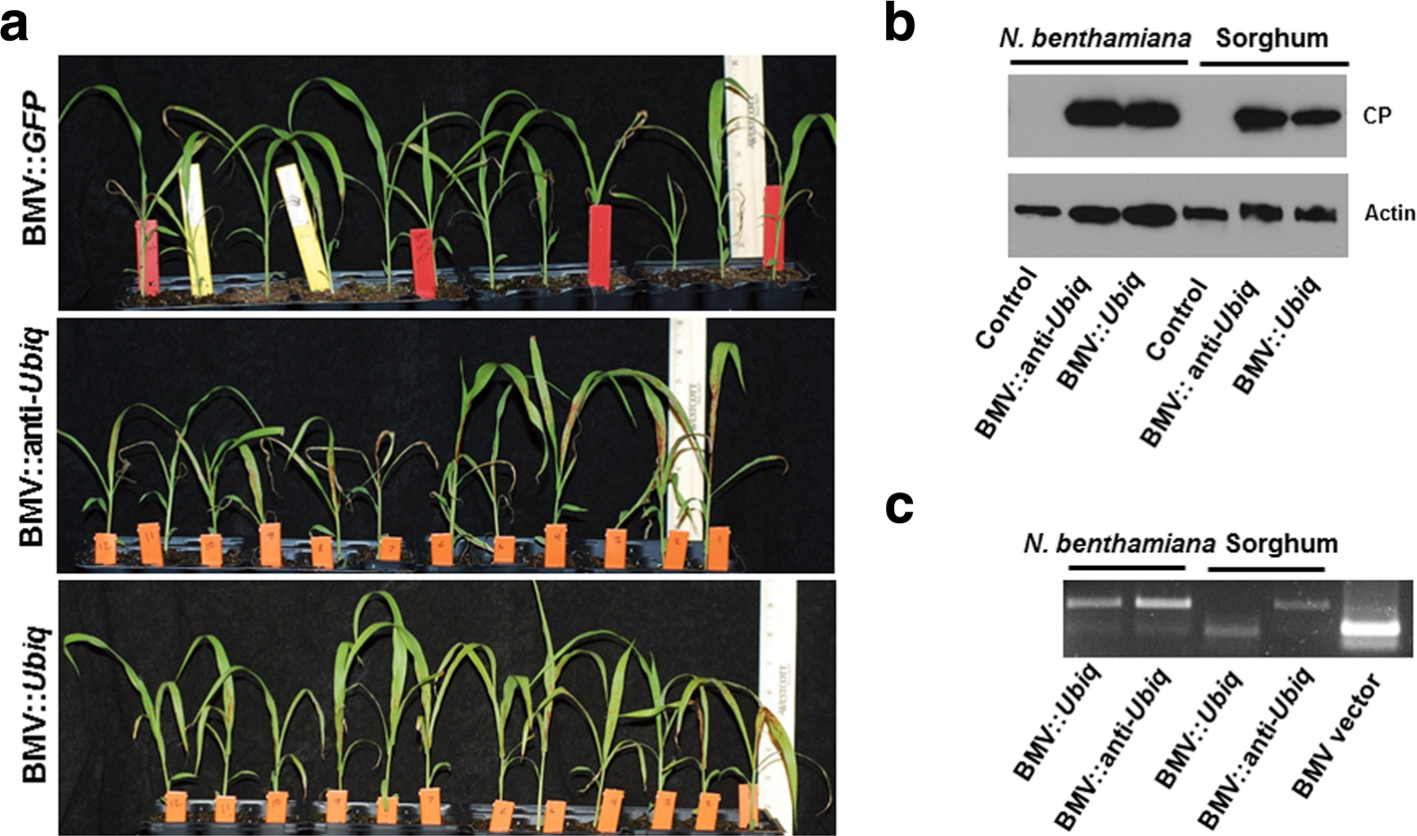
BMV VIGS is more efficient when an anti-sense strand of the gene fragment is used for silencing. Two-week old sorghum plants were rub inoculated with BMV containing sap of *N. benthamiana*. **a** The BMV::*Ubiq* and BMV::anti-*Ubiq* infected plants had a varying degree of *Ubiq* silencing. The extent of silencing was more severe in the BMV::anti-*Ubiq* infected plants. The experiment was repeated three times with similar results. **b** BMV level was analyzed by western blot using an antibody against BMV coat protein. BMV level was more in the sorghum plants infected with BMV: anti-*Ubiq* compared to BMV::*Ubiq*. **c** RT-PCR was performed to detect the presence of gene fragment in the BMV RNA3 using primers flanking the insert. The BMV with insert was present in the sorghum infected with BMV::anti-*Ubiq* but not detectable in sorghum infected with BMV::*Ubiq*. For RT-PCR and western blot, leaves of three *N. benthamiana* plants or 20 sorghum plants per sample were pooled to extract the virus

Sorghum plants infected with either BMV::*Ubiq* (sense strand) or BMV::anti-*Ubiq* (antisense strand) developed characteristic brown coloration due to *Ubiquitin* silencing (Fig. 4a). However, the brown coloration was more widespread in the leaves of BMV::anti-*Ubiq* inoculated compared to BMV::*Ubiq* inoculated sorghum (Fig. 4a). Browning was observed in 100% of BMV::anti-*Ubiq* infected plants whereas only 73% in BMV::*Ubiq* infected plants. We further quantified the level of BMV in the infected *N. benthamiana* that was used to inoculate sorghum plants (Fig. 4b). Antibody specific to the coat protein of BMV was used in the western blot to quantify the virus. When normalized to Actin protein, the levels of BMV was relatively more in the BMV::*Ubiq* infected compared to the BMV::anti-*Ubiq* infected *N. benthamiana* plants (Fig. 4b and Additional file 1: Figure S1). Similar amounts of *N. benthamiana* leaf sap were used to inoculate sorghum. However, BMV amount in the BMV::anti-*Ubiq* inoculated sorghum plants was slightly (~25% after normalization to control) more compared to BMV::*Ubiq* inoculated plants (Fig. 4b and Additional file 1: Figure S1).

Further, the presence of the *Ubiq* gene fragment (insert) in the BMV VIGS vector isolated from *N. benthamiana* and sorghum inoculated plants was tested. RT-PCR was performed using primers specific to BMV RNA 3 flanking the insertion site. In *N. benthamiana*, the virus with insert was more compared to the virus without the insert in both BMV::*Ubiq* and BMV::anti-*Ubiq* infected plants. In sorghum, the virus with insert was more in the BMV::anti-*Ubiq* inoculated plants when compared to BMV::*Ubiq* inoculated plants. Interestingly, the virus without the insert was more in the sorghum plants inoculated with BMV::*Ubiq*. These results indicate that in sorghum, antisense strand of *Ubiq* fragment is more stable than sense strand in BMV VIGS vector. However, more experiments with other gene fragments will be needed to conclude that antisense strand will be more efficient for VIGS in sorghum.

### BTx623 is a good sorghum variety for BMV-based VIGS

As mentioned above, one of the important requirements for VIGS is the ability of the virus to infect and multiply in a given plant. Frequency of sorghum infection by BMV, especially at 22 ^o^C and above, was quite low. We speculated that variations in recalcitrance to BMV infection might exist among different genotypes/varieties of sorghum. Therefore, 67 sorghum genotypes/cultivars were screened to identify a highly susceptible genotype/variety to BMV. Two-week old sorghum plants were rub inoculated with BMV::*GFP*. Three days before rub inoculation, the plants were placed in a growth chamber at 18 °C and 12/12 h light/dark cycle. After inoculation, the plants were maintained at 18 °C for four weeks. At four weeks post-inoculation, the systemic leaves (two leaves above the rub-inoculated leaf) were examined for white stripes (BMV infection symptom). The percentage of inoculated plants showing infection symptoms was recorded (Additional file 2: Table S1). Six genotypes/cultivars with varying degrees of susceptibility to BMV were selected for re-examination by inoculating them with BMV::*GFP* and BMV::anti-*Ubiq* (Table 1). Based on the screening, BTx623 was identified as the most susceptible variety for both the constructs of BMV used (Table 1).

**Table 1:** A representative list of sorghum genotypes/varieties showing varying degrees of susceptibility to BMV

*Sorghum bicolor* genotype^a^	Percent of plants showing disease phenotype after BMV challenge^b^	
	BMV::*GFP*	BMV::anti-*Ubiq*
BTx623	100.00	100.00
Rio	100.00	83.33
Topper 76	80.00	61.54
PI 641817	54.77	50.00
N122B	41.67	33.33
N588	18.18	16.67

^a^More than ten plants of each line were used for screening.

^b^Two weeks old sorghum plants were rub inoculated with BMV::*GFP* or BMV::anti-*Ubiq* and plants showing BMV infection symptom were scored three weeks after inoculation.

### Non-uniform silencing in sorghum correlates with uneven distribution of BMV in the infected leaf

**Fig. 5:**
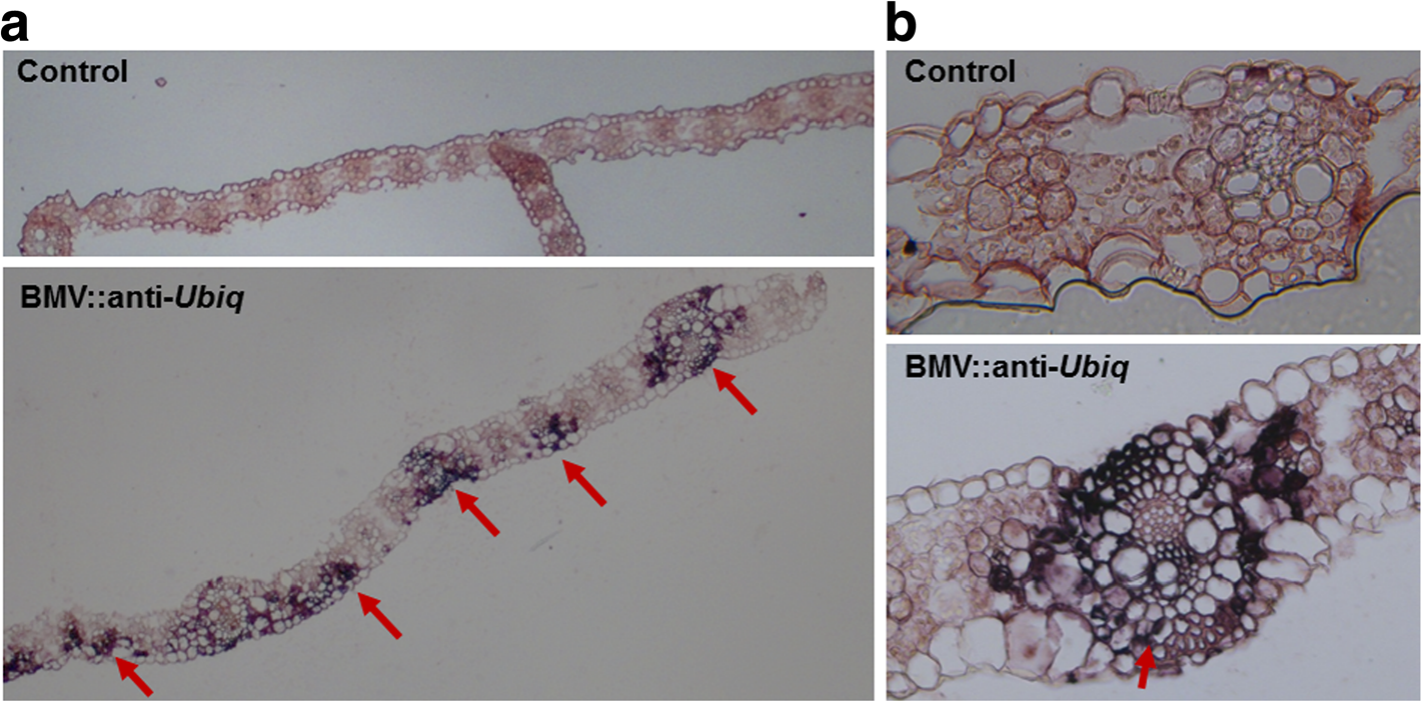
*In situ* hybridization shows the uneven spread of BMV virus in sorghum. *In situ* hybridization probe was designed to hybridize the RNA encoding BMV coat protein. The purple pigment (red arrows) indicates the presence of the virus near veins of the infected leaf. The absence of purple color in the uninfected control sorghum plant indicate a lack of virus. The image was taken with a Nikon TE300 microscope. **a** Sorghum leaf segment observed by the 10X magnification of the Nikon TE300 microscope. **b** The sorghum leaf segment observed by the 20X magnification of the microscope. A similar pattern was seen in two biological replicates

Uniform silencing of a gene is observed in leaves of *N. benthamiana* by *Tobacco rattle virus* -based VIGS [34]; in leaves of soybean, *N. benthamiana*, and pea (*Pisum sativum*) by *Apple latent spherical virus* (ALSV) -based VIGS [39]; and in leaves of barley by BSMV based VIGS [29]. However, uniform silencing of *Ubiq* in sorghum by BMV based VIGS vector is rare. To determine the biological reason for this phenomenon, the presence of BMV in the cells of the infected leaf was observed using a probe designed to hybridize the RNA encoding the coat protein of the virus. The *in situ* hybridization results suggested that the viral RNA was distributed unevenly throughout the leaf with more concentration around the vascular tissue (Fig. 5a). However, the viral RNA was not present in all the cells of infected vascular tissue (Fig. 5b). These results suggest that the non-uniform silencing in sorghum leaves upon BMV infection is due to the uneven spread of the virus in the leaves.

### VIGS can be achieved in sorghum floret

**Fig. 6:**
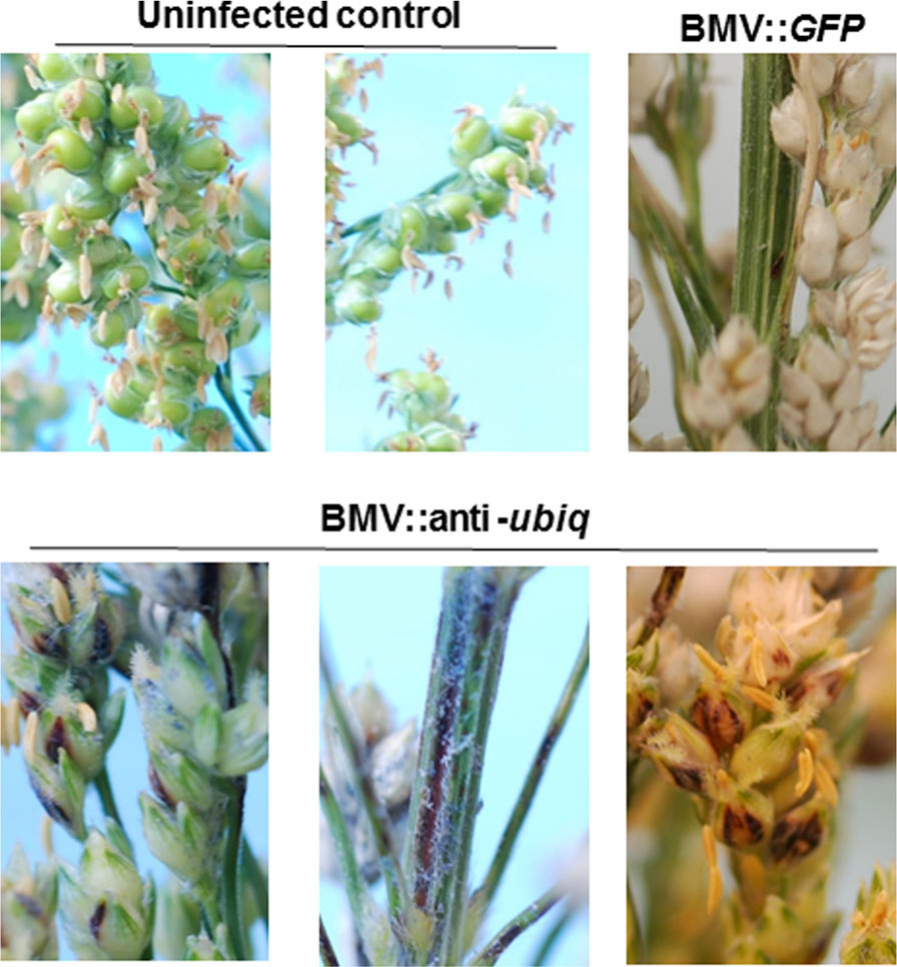
Silencing of *Ubiquitin* gene in sorghum inflorescence. The BMV::anti-*Ubiq* construct was rub inoculated on the inflorescence of the sorghum plants that was kept at 18°C for two days. The brown pigmentation due to *Ubiq* silencing was observed on the floret, rachilla, and rachis of inoculated plants. The brown pigment region increased over time. Three individual sorghum plants were inoculated and showed similar results. The experiment was performed twice with same results

Determination of gene function in the seed and embryo is crucial to study some traits such as yield. VIGS has been used to study gene function in the inflorescence of soybean and wheat [27, 40, 41]. Silencing of a sorghum gene in the inflorescence cannot be achieved by inoculating leaves with BMV-VIGS construct because VIGS is transient. Therefore, a technique was developed to silence genes in the sorghum inflorescence tissue. Sorghum variety Topper 76 was selected as it is susceptible to BMV and also smaller in size that makes it convenient for inoculation and to grow in the greenhouse. Sorghum inflorescence was rub-inoculated with the sap of *N. benthamiana* that was infected with BMV::anti-*Ubiq* construct. The inoculated plants were kept at 18 °C in the dark and humid environment for two days before transferring to a greenhouse with 22 °C temperature. Keeping plants longer in 18 °C chamber with 70% humidity at the flowering stage resulted in leaf damage. The browning due to *Ubiq* gene silencing was clearly observed in the sorghum floret, rachilla, and rachis (Fig. 6). Initially, silencing was observed in a small area but over time the area of inflorescence showing silencing phenotype increased. These preliminary results suggest that BMV-mediated VIGS can be used to silence genes in reproductive tissue of sorghum.

### BSMV-mediated VIGS was not observed in sorghum line BTx623

**Fig. 7:**
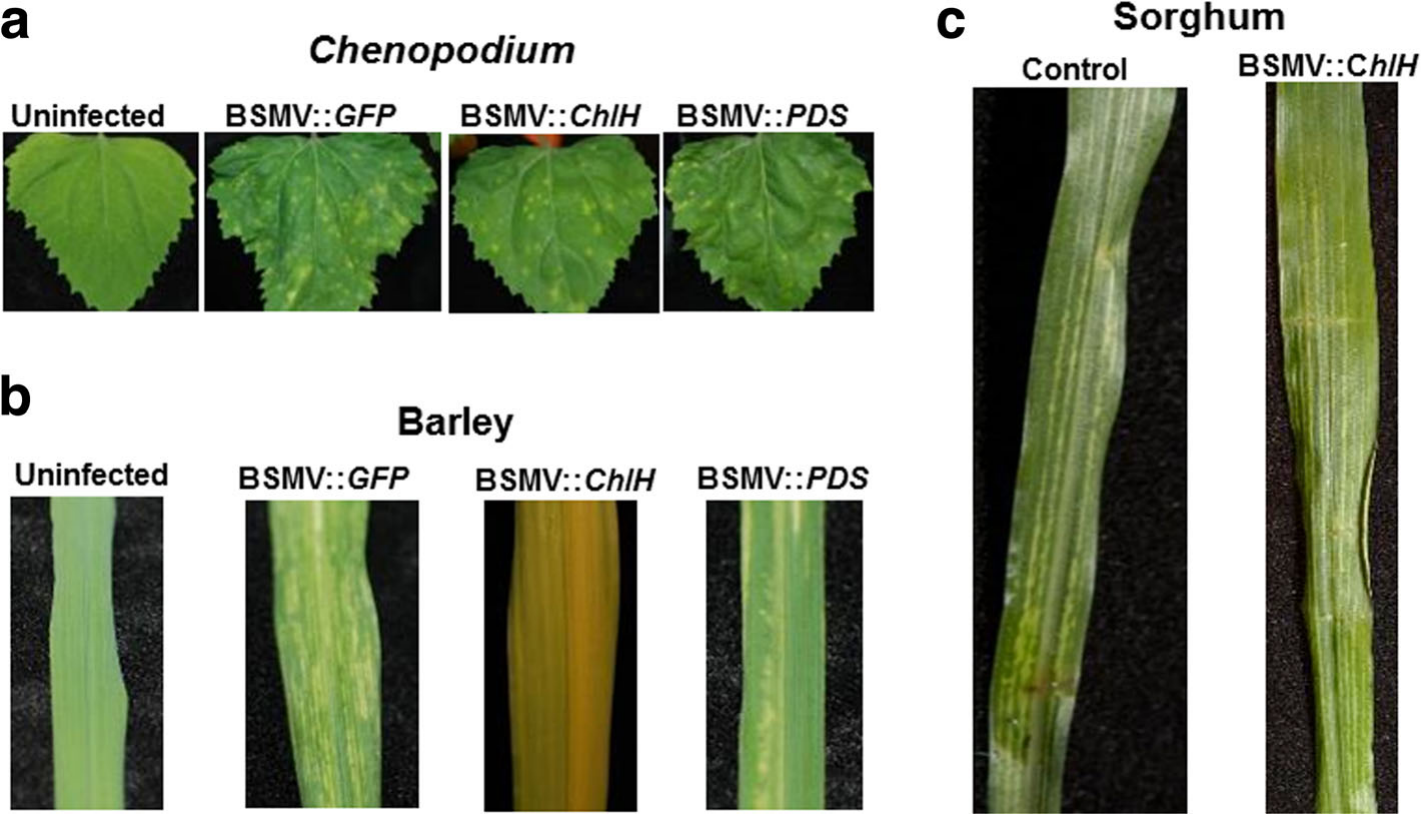
Sorghum plants are resistant to BSMV infection. Sap from the BSMV inoculated *N. benthamiana* plants was used to rub inoculate *Chenopodium*, barley, and sorghum. **a** The *Chenopodium* plants showed lesions suggesting that the virus is infectious. **b** The white stripes in the systemic leaves of inoculated barley plants suggest successful infection by the virus at 22 °C. The yellowing of the BSMV:: *ChlH* infected plants indicate successful silencing of the *ChlH* gene in barley. **c** Sorghum plants kept at either 20 °C or 18 °C had no visible symptoms even 40 days after inoculation with BSMV. White stripes observed on leaves of both uninoculated control and BSMV inoculated sorghum plants is not due to virus infection. The experiment was performed with three barley, two *Chenopodium*, and 20 sorghum plants. The experiment was repeated twice. Similar results were observed in all the replicates of both the experiments

In addition to BMV, *Barley stripe mosaic virus* (BSMV) was also tested as a potential VIGS vector for sorghum. BSMV is popularly used to silence genes in barley and wheat [29]. *Agrobacterium*-based [29] and *in vitro* viral RNA synthesis based [38] BSMV-VIGS systems are available. We used *Agrobacterium*-based BSMV-VIGS system to silence *ChlH*, *PDS*, and *GFP* (control) in sorghum (BTx623). The BSMV genome (α–RNA, β-RNA, and γ–RNA) is cloned into three different *Agrobacterium* vectors. A fragment of plant gene was inserted into γ–RNA as described earlier [29]. All the three *Agrobacterium* strains containing α–RNA, β-RNA, and γ–RNA were grown to 1.5 O.D_600_, mixed in equal amounts and infiltrated into three weeks old *N. benthamiana* leaves for viral multiplication. The infected *N. benthamiana* leaves were harvested after four days and were used to extract sap for infecting *Chenopodium*, barley and sorghum plants. All the BSMV-VIGS constructs generated were able to infect *Chenopodium* and barley successfully (Fig. 7a, and 7b). The leaves of barley infected with BSMV::*ChlH* turned yellow, a phenotype commonly observed upon silencing of *ChlH* gene (Fig. 7b; [29]). However, we could not observe any viral symptoms in BTx623 cultivar of sorghum plants inoculated with BSMV. Non-inoculated and inoculated sorghum plants kept at 18 °C looked similar even 40 days after BSMV inoculation (Fig. 7c). Therefore, we concluded that BSMV based VIGS is not efficient in BTx623 cultivar of sorghum.

## Discussion

The annotated sorghum BTx623 genome has 34,211 protein-encoding loci as reported in Phytozome 11 (https://phytozome.jgi.doe.gov). A robust method is needed to study gene function to improve agronomic traits. Unlike Arabidopsis and rice, stable T-DNA insertion mutants are not available in sorghum. Genetic manipulation of sorghum to generate knockdown/knockout or overexpressor lines is cumbersome requiring several months and a tissue culture facility [42]. VIGS is an alternative method for the functional study of a gene in sorghum. In the present study, we report an improved method of VIGS in sorghum.

The first step for successful VIGS is to infect a plant with the virus. At ambient greenhouse growth condition (22 °C) the infectivity of BMV on sorghum occurs at low frequency (Fig. 2a and 2b). Many studies have reported increased infectivity of virus on plants and animals at a lower temperature than the ambient temperature [43, 44]. Increased viral infectivity at a lower temperature is suggested to be due to reduced antiviral defense of the host [43–45]. In addition, lower temperatures have been shown to enhance VIGS phenotype in several dicotyledonous plant species while higher temperature have been shown to increase viral genome silencing [39, 44, 46–48]. In addition, lower light intensity (<300 μE/m2/s) and temperature (25 °C) have more efficient systemic silencing compared to higher light intensity (≥ 450 μE/m2 /s) and temperature (30 °C) [49]. Our study showed that higher frequency of infection by BMV could be achieved by infecting sorghum plants kept at 18 °C. Similarly, in wheat, BSMV based VIGS showed better silencing phenotype at lower temperatures of 18-22 ^o^C [50]. However, several reports in monocotyledonous plants have shown that higher temperature increases viral infection. In barley, BSMV-mediated VIGS provided better results at 20-24 ^o^C when compared to 16 or 28 ^o^C [38]. In addition, Ding et al. [51] showed that in barley at lower temperatures (24/20 °C, day/night), the BMV virus infected and accumulated mainly in and near vascular cells and limited invasion of vascular tissue was observed. These results suggest that the effect of temperature on VIGS varies among plant-virus combinations and therefore it is important to optimize temperature conditions for every VIGS vector-plant combination studied.

The photobleaching and yellowing of leaves due to *PDS* and *ChlH* silencing, respectively, is used as an indicator of VIGS in dicotyledon and monocotyledon plants [34]. Surprisingly, *PDS* or *ChlH* gene silencing in sorghum by BMV-mediated VIGS did not result in distinctly visible phenotype in sorghum. However, white/yellow stripes on the leaves due to virus infection were visible (Fig. 3a). *PDS* or *ChlH* silencing is therefore not a suitable VIGS marker in sorghum. We propose several reasons why *PDS* and *ChlH* are not suitable VIGS markers in sorghum. 1) The white phenotype due to *PDS* silencing is similar to virus infection due to BMV infection, 2) the *PDS/ChlH* silencing phenotype is also similar to nitrogen deficiency symptom in sorghum, 3) sorghum is probably tolerant to reduced levels of PDS and ChlH proteins and therefore doesn’t show any visible symptoms.

The housekeeping gene *Ubiq* is essential for cellular function by targeting proteins for degradation. Therefore, *Ubiq* gene silencing is expected to cause significant cellular dysfunction leading to cell death, which will be distinctly visible. Partially reducing *Ubiq* expression level by transient gene silencing in barley lead to cell death [35]. Consistent with these results, our study showed that *Ubiq* silencing in sorghum by BMV-mediated VIGS showed a clear browning (cell death) phenotype (Fig. 3a).

Higher efficiency of gene silencing using antisense strand rather than the sense strand of *Ubiq* during VIGS was observed in our study. BMV is a positive (+) strand RNA virus of family *Bromoviridae*. In infected host cells, the + strand RNA viruses produce 10 to 100 fold more + strand genomic RNA than the negative (-) strand RNA [52]. Consistently, BMV produces more + strand compared to – strand [53, 54]. Higher + strand of virus lead to a greater level of viral siRNA from the + strand as observed in plants or animals infected with + RNA viruses [52, 55–58]. The higher silencing efficiency of the antisense strand of the gene might be due to generation of more siRNA. Also, in sorghum, we detected more retention of the antisense strand than the sense strand in the recombinant BMV. Further, more virus was present in sorghum leaves when the gene fragment was in antisense strand than in sense strand. The cumulative effect of these factors might result in higher VIGS efficiency when antisense strand is used.

VIGS is used to study the function of a gene in the inflorescence of plants. For example, a soybean (*Glycine max*) seed coat color was modified by silencing a gene using *Cucumber mosaic virus* based VIGS [40]. Similarly, a *PDS* gene was silenced in immature pod and seed coat of soybean using ALSV based VIGS by inoculating the first leaf or cotyledon [41]. BSMV based VIGS was used to silence a gene in wheat flowers by inoculating the inflorescence since inoculating seedling was not sufficient to silence a gene in flowers [27]. When the leaves of sorghum seedlings were inoculated with BMV based VIGS, silencing was observed only in up to three new leaves that emerged after inoculation. However, upon infection of the inflorescence with BMV::anti-*Ubiq* it resulted in the browning of floret, rachilla, and rachis of sorghum (Fig. 6). This is the first report of gene silencing in sorghum inflorescence using VIGS.

## Conclusion

Several VIGS vectors are available to silence genes in monocots. At least two studies report the use of BMV-based VIGS to silence genes in sorghum [25, 26]. The silencing efficiency up to 50% was achieved in few plants (low frequency) using this method [25, 26]. Here, we tested several parameters to develop a more efficient BMV based VIGS for sorghum. The virus was multiplied in *N. benthamiana* as done in maize VIGS studies with this vector [33] and unlike in Martin et al., [26] and Biruma et al., [25], where BMV viral mRNA was synthesized *in vitro* to directly infect sorghum plants. We found three factors that significantly increased VIGS efficiency and frequency in sorghum. 1) Using antisense strand of a gene to develop the VIGS construct instead of sense construct. 2) Keeping the sorghum plants at a lower temperature (18 °C) before and after virus inoculation. 3) Using sorghum variety (eg., BTx623) that allows more BMV multiplication. Further, we identified an appropriate marker gene (*Ubiq*) that shows clear visible symptoms when silenced. In addition to an improved method for VIGS in sorghum, we also showed that we could silence genes in the reproductive tissue of sorghum by using BMV-based VIGS. An efficient BMV-based VIGS will give a significant boost to gene functional analysis related studies in sorghum.

## Methods

### Plant growth, inoculation and disease assessment

Sorghum, barley, *Chenopodium,* and *N. benthamiana* plants were grown in a greenhouse at 22 °C day and night temperature. All plants were grown in the Metromix 360. Leaves of three weeks old *N. benthamiana* plants were infiltarted with disarmed *Agrobacterium tumefaciens* strain GV2260 containing the recombinant BMV vectors within the T-DNA using a needless syringe. Inoculated (as described below) barley, *Chenopodium*, and *N. benthamiana* plants were kept in the greenhouse with 16 h photoperiod and 22 °C temperature. Sorghum plants were maintained in a growth chamber at 18 °C, 12 h photoperiod, 70 % humidity, and 150-200 μmolm^-2^s^-1^ light intensity 3 days before inoculation and 3 weeks post inoculation. The BMV infection symptom was assessed by counting the plants with white stripes in the second leaves above the infected leaves at 4 weeks after rub inoculation.

### Developing BMV VIGS constructs

The RNA from the 3 weeks old sorghum plants was used to generate cDNA to PCR amplify gene fragments for VIGS construct generation. The 5´end of the primers was designed to have *Nco*I or *Avr*II restriction enzyme sites for restriction digestion and cloning into BMV-VIGS vector [15, 33]. The primer sequences used for the experiment are listed in Additional file 3: Table S2. The size of the insert was from 200 to 400 base pairs. After cloning the insert into the vector, BMV3 F primer (Additional file 3: Table S2) was used for sequencing. The plasmid with the insert was transformed into *A. tumefaciens* strain GV2260.

### Plant inoculation with BMV

Two *Agrobacterium* strains, one with RNA1 and RNA2 of BMV and another with RNA3 of the BMV genome were grown overnight in LB medium. The strains were centrifuged and resuspended to 1.5 OD_600_ in 10 mM MES (pH 5.8) and 100 nM acetosyringone. An equal amount of two *Agrobacterium* strains were mixed and kept in a shaker at room temperature for 3 h in the dark. The *Agrobacterium* cocktail was syringe (needless) infiltrated into fully open leaves of *N. benthamiana* grown in a greenhouse at 22 °C. *N. benthamiana* leaves were harvested 4 days after infiltration and used to prepare sap as described below or were frozen in liquid nitrogen and stored at -80 °C for future use.

The inoculation method is schematically represented in Fig. 1. Sorghum plants were grown in a greenhouse at 22 °C for 12 days (four-leaf stage). The young plants were then transferred to a growth chamber at 18 °C and 12 h/12 h light/dark cycle for three days. The sap for rub-inoculation was prepared by grinding 2 g of infected *N. benthamiana* leaf in 1 ml 10 mM potassium phosphate buffer (pH 6.8) and 100 mg of carborundum abrasive. The sap is rubbed on two to three bottom leaves of sorghum. Inoculated plants were covered with a dome to maintain high humidity and kept in the dark for two days at 18 °C. Inoculated plants were then transferred to 18 °C growth chamber with 12 h/12 h light/dark cycle. Similar to sorghum, *Chenopodium* and barley plants were also inoculated with the sap as described above for sorghum, except that the plants were kept at 22 °C. BSMV inoculation was also done in a similar fashion as BMV.

### Quantitative RT-PCR

Total plant RNA was extracted using a Qiagen RNAeasy kit (www.qiagen.com). The RNA was treated with RNAse-free DNAse I. Superscript III was used to synthesize the first strand of the cDNA and oligo (dT) primer. Quantitative RT-PCR was performed using 10-fold diluted cDNA, SYBR green Real-time PCR Master Mix (www.thermofisher.com), and ABI PRISM 7500 (Applied Biosystems, NY, USA). The transcript level of a gene was normalized with *SbActin*. Student's *t*-test determined the statistically significant difference between the expression level in the silenced and control plants.

### Virus extraction and RT-PCR

For virus extraction, leaves of *N. benthamiana* were harvested 4 days after inoculation and leaves of sorghum were harvested three weeks post inoculation. BMV was extracted by the polyethylene glycol precipitation method [15]. Phenol/chloroform was used to extract RNA from the purified virus [15]. The first strand of the cDNA was synthesized using Superscript III and random primers. PCR using BMV3 forward and reverse primers (Additional file 3: Table S2) that flank the insert DNA in the RNA3 of the virus was performed.

### BMV quantification

Total protein was extracted from the leaves of infected *N. benthamiana* and sorghum using extraction buffer containing 1 mM MgCl2, 220 mM Tris/HCl pH 7.4, 50 mM KCl, 250 mM sucrose, and 10 mM β– mercaptoethanol. An equal amount (2 ug) of extracted protein was separated using SDS-PAGE in 12.5% gels and then subjected to western blot using an antibody specific to BMV coat protein [51].

### *In situ* hybridization

The BMV RNA3-Insitu-L/R primer pairs (Additional file 3: Table S2) were used in PCR for probe preparation to amplify a 362-bp of the RNA3 of BMV. The PCR probe was labeled with Digoxigenin-11-UTP (Sigma-Aldrich Co. LLC, Mo, USA). Long’s protocol (http://www.its.caltech.edu/~plantlab/protocols/insitu.pdf) was used for tissue preparation. Robot GenePaint system (Tecan) was used for pre-hybridization, hybridization, and washing [59]. The section was imaged using the Nikon Eclipse TE 300 (Nikon Inc. NY, USA).

## Additional files

Additional file 1: **Figure S1.** BMV capsid protein quantification. BMV level was analyzed by western blot using an antibody against BMV coat protein. The capsid protein was normalized with Actin protein of the plants. In *N. benthamiana*, the BMV level was more in the BMV:: *Ubiq* infected plant compared to BMV:: anti-*Ubiq* infected plant. In sorghum, BMV level is similar in both BMV:: anti-*Ubiq* and BMV:: *Ubiq* infected plants. (PDF 63 kb)

Additional file 2: **Table S1.** List of sorghum genotypes/varieties showing BMV infection. (XLSX 11 kb)

Additional file 3: **Table S2.** List of primers used in this study. (XLSX 12 kb)

## Abbreviations

VIGS: Virus-induced gene silencing
BMV: *Brome mosaic virus*
BSMV: *Barley stripe mosaic virus*
Ubiq: Ubiquitin
ChlH: Magnesium chelatase subunit H
PDS: Phytoene desaturase
SiRNA: Small interfering RNA
