# Supplementary material for: An efficient and improved method for virus-induced gene silencing in sorghum
Source: BMC Plant Biol. 2018 Jun 18;18:123. doi: 10.1186/s12870-018-1344-z (PMC6006947; doi:10.1186/s12870-018-1344-z)
Supplement: Supplementary file 1 — Figure S1. BMV capsid protein quantification. BMV level was analyzed by western blot using an antibody against BMV coat protein. The capsid protein was normalized with Actin protein of the plants. In N. benthamiana, the BMV level was more in the BMV:: Ubiq infected plant compared to BMV:: anti-Ubiq infected plant. In sorghum, BMV level is similar in both BMV:: anti-Ubiq and BMV:: Ubiq infected plants. (PDF 63 kb) [file 12870_2018_1344_MOESM1_ESM.pdf]

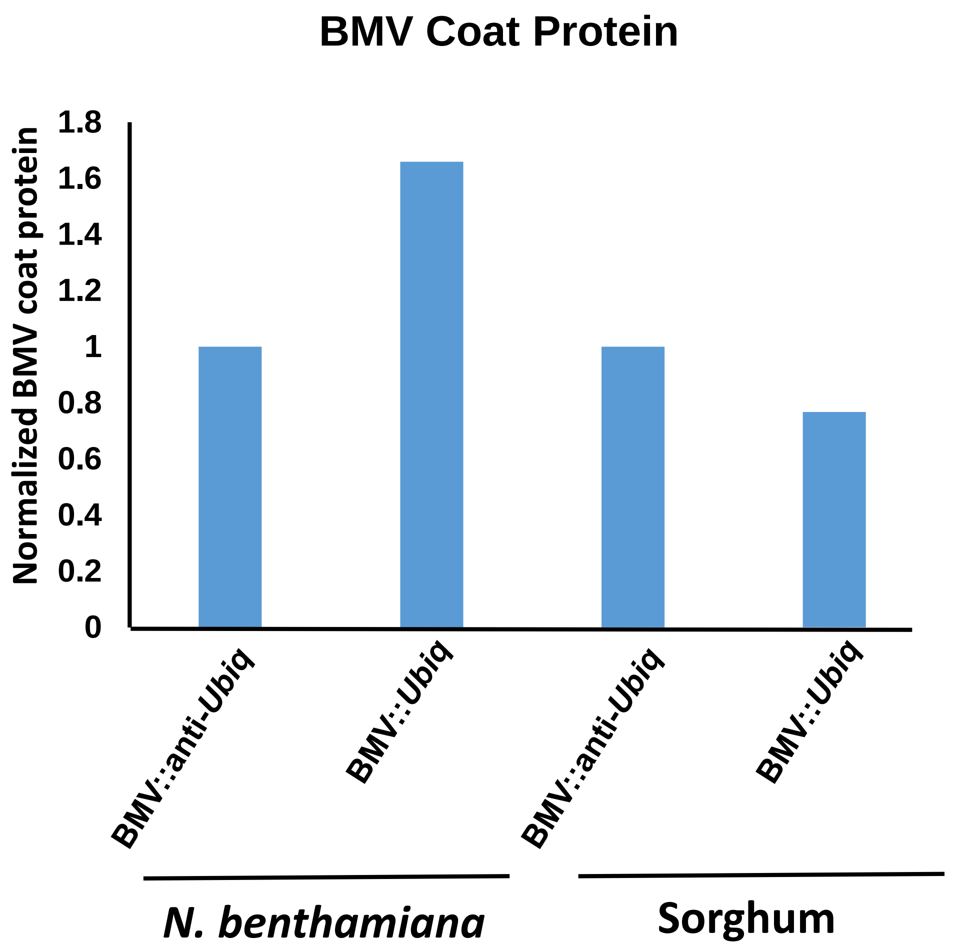

Supplementary Figure S1. BMV capsid protein quantification. BMV level was analyzed by western blot using an antibody against BMV coat protein. The capsid protein was normalized with Actin protein of the plants. In *N. benthamiana*, the BMV level was more in the BMV::*Ubiq* infected plant compared to BMV::a*nti-Ubiq* infected plant. In sorghum, BMV level is similar in both BMV::anti-*Ubiq* and BMV::*Ubiq* infected plants.
